# Potential for functional divergence in ectomycorrhizal fungal communities across a precipitation gradient

**DOI:** 10.1093/ismeco/ycae031

**Published:** 2024-03-04

**Authors:** Peter T Pellitier, Michael Van Nuland, Asaf Salamov, Igor V Grigoriev, Kabir G Peay

**Affiliations:** Department of Biology, Stanford University, Stanford CA 94305, United States; Department of Earth System Science, Stanford University, Stanford CA 94305, United States; Department of Biology, Stanford University, Stanford CA 94305, United States; Society for the Protection of Underground Networks, SPUN, Dover, DE 19901, United States; Department of Energy Joint Genome Institute, Lawrence Berkeley National Laboratory, Berkeley, CA 94720 United States; Department of Energy Joint Genome Institute, Lawrence Berkeley National Laboratory, Berkeley, CA 94720 United States; Department of Plant and Microbial Biology, University of California Berkeley, Berkeley, CA 94720, United States; Department of Biology, Stanford University, Stanford CA 94305, United States; Department of Earth System Science, Stanford University, Stanford CA 94305, United States

**Keywords:** ectomycorrhizal fungi, metagenomics, drought, gradient, profiling, community assembly

## Abstract

Functional traits influence the assembly of microbial communities, but identifying these traits in the environment has remained challenging. We studied ectomycorrhizal fungal (EMF) communities inhabiting *Populus trichocarpa* roots distributed across a precipitation gradient in the Pacific Northwest, USA. We profiled these communities using taxonomic (meta-barcoding) and functional (metagenomic) approaches. We hypothesized that genes involved in fungal drought-stress tolerance and fungal mediated plant water uptake would be most abundant in drier soils. We were unable to detect support for this hypothesis; instead, the abundance of genes involved in melanin synthesis, hydrophobins, aquaporins, trehalose-synthases, and other gene families exhibited no significant shifts across the gradient. Finally, we studied variation in sequence homology for certain genes, finding that fungal communities in dry soils are composed of distinct aquaporin and hydrophobin gene sequences. Altogether, our results suggest that while EMF communities exhibit significant compositional shifts across this gradient, coupled functional turnover, at least as inferred using community metagenomics is limited. Accordingly, the consequences of these distinct EMF communities on plant water uptake remain critically unknown, and future studies targeting the expression of genes involved in drought stress tolerance are required.

## Introduction

Identifying functional traits that mediate the distribution and functioning of microbial communities represents an urgent area of research [1]. This is because microbial trait distributions can promote understanding of the role of microbes in key biogeochemical transformations, such as those required for adaptation to altered precipitation regimes. Historically, the analysis of microbial traits has largely relied on morphological or process-based measurements. The abundance of molecular genes can serve as proxies for microbial functioning and traits, and they have the advantage of being readily studied using whole-community metagenomic profiling [2, 3].

Ectomycorrhizal fungi (EMF) are dominant microbial members of forest ecosystems. Well-known for their role in plant nutrient uptake, EMF may also improve plant drought stress tolerance via specialized water transporters that increase root hydraulic conductivity [4, 5] and by extending the surface area of plant roots. Through their role in plant water uptake, EMF support the evolution of drought tolerance in host plants [6]. However, the extent to which EMF serve to extend plant drought tolerance and increase plant water uptake under field conditions has remained inconclusive [4].

In light of laboratory evidence that EMF influence plant water relations, widespread observations of EMF community turnover across precipitation gradients is notable [7]. Two coupled processes could generate turnover in EMF composition and function across precipitation gradients. First, these patterns could result from variation in inherent EMF physiological tolerance to soil water availability or conditions which are modified by increased water availability. Secondly, plant water demand may influence EMF community composition if plants reward EMF that transfer greater water resources [8]. Such non-mutually exclusive processes would result in coupled compositional and functional trait turnover, whereby traits involved in water acquisition and fungal drought tolerance would exhibit the greatest turnover [9].

Shifts in gene counts per genome, or gene counts measured at the community level, represent tractable molecular approaches to study shifts in microbial trait profiles [2]. Metagenomic measures of microbial genes have been used to identify genes that could serve to determine the suitability of organisms to the local environment [2, 3]. This approach is conceptually similar to analysing plant leaf or root traits along ecological gradients where coupled shifts are considered indicative of trait-based environmental filtering [9].

**Figure 1:**
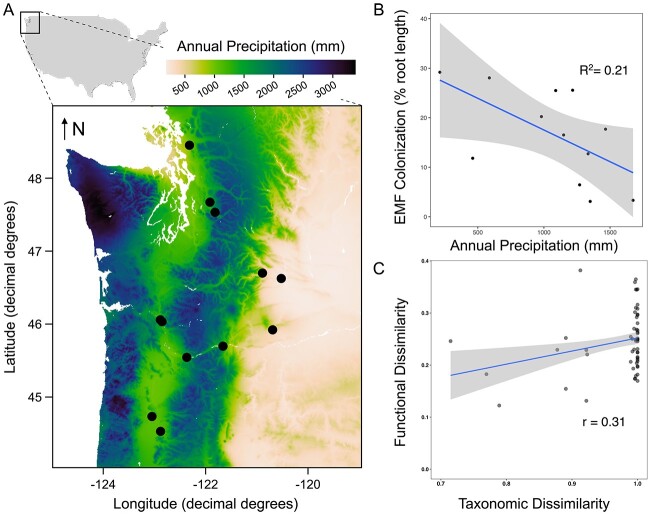
(A). Sites across the Pacific Northwest (USA), where ectomycorrhizal fungal (EMF) communities associated with *Populus trichocarpa* were sampled. (B) EMF colonized root length was inversely correlated with mean annual precipitation (MAP; *P =* .04; *R*^2^ = 0.21). (C) Significant taxonomic and functional coupling for EMF communities, as measured using counts of Pfam annotated genes (Bray–Curtis distances). Taxonomic dissimilarity is measured using fungal amplicon sequence variants (ASV). For (B and C), Lines represent linear splines with 95% confidence intervals.

In the current study, we studied EMF communities inhabiting the roots of *Populus trichocarpa* distributed across an ecosystem-scale gradient of water availability in the Pacific Northwest, USA (Fig. 1A). *P. trichocarpa* is a widespread and important pioneer tree species in riparian habitats; we studied this species as a standardized host to remove the potentially confounding effects of host differences on EMF communities. We tested the hypothesis that EMF communities exhibit coupled taxonomic and functional shifts across a precipitation gradient, and that EMF communities inhabiting drier soils are enriched in genes that promote drought stress tolerance and host plant water uptake. We focused on a core set of gene families which previous work identified as most likely involved in fungal drought stress tolerance and water uptake. Due to the relatively small sample size, we wanted to test *a priori* hypotheses regarding specific gene families rather than focusing on global scale genome analysis. I we focused on core gene families such as fungal hydrophobins, aquaporins, melanins, and trehalose synthases, collectively some of the best characterized genes related to drought tolerance for fungi [10–13]. Fungal aquaporins are thought to play a critical role in transmembrane water transfer to host roots and may beneficially influence root water uptake [5]. Melanin is a component of fungal cell wall and has been shown to improve drought stress tolerance by reducing osmotic stress [14]. Fungal hydrophobins are small amphiphilic molecules that coat hyphae, serving to facilitate growth across air-filled soil pores. Finally, we also studied certain carbohydrate active enzymes (CAZy), which could be involved in cell wall remodeling and transmembrane water transfer [13].

## Results and discussion

In July and August of 2017, we conducted a field survey of root samples from 12 forest sites dominated by *P. trichocarpa* [15]. These sites represent a subset of a continental scale sampling network [16]. The 12 field sites differ markedly in mean annual precipitation (MAP: range = 213–1674 mm yr^−1^; Fig. 1a), and we used MAP as a coarse proxy for soil water availability. EMF root-tip colonization (% root-length colonized: grid-intersect method [17]) ranged from approximately 4–29%, and colonization was inversely correlated with mean annual precipitation (MAP; *P =* 0.04; Fig. 1b). We characterized EMF communities in *P. trichocarpa* using metabarcoding of the ITS1 region of rDNA. Soil properties such as pH, and soil carbon and nitrogen were also measured from the sampled soil cores (Supplementary Methods).

The relative abundance of Basidiomycete and Ascomycete EMF were invariant across the sampled gradient (Supplementary Figure 1). However, at finer taxonomic scales, EMF communities exhibited significant compositional turnover. Community dissimilarity as measured using fungal ASVs, was primarily associated with variation in soil pH (*P =* 0.01), and marginally with MAP (*P* = 0.06; PERMANOVA; Supplementary Table 1; Supplementary Figure 2), but non-significantly by other soil chemical parameters such as soil carbon, or nitrogen release. It should be noted that soil pH and MAP were significantly correlated, with drier sites being more alkaline (R^2^ = 0.25, P < 0.001); this correlation is often observed, in part because increased precipitation reduces the availability of buffering ions (i.e. carbonates; [18]). Overall, EMF genera such as *Tuber* and *Wilcoxina* were dominant in drier sites, whereas *Scleroderma* and *Geopora* were most abundant in wetter sites (Supplementary Figure 3).

The same DNA pool was used for the construction of metagenomic libraries, sequenced using Illumina NovaSeq. We employed the JGI IMG pipeline to filter and annotate fungal reads to Pfam domains, whilst removing plant and bacterial sequences [19] (Supplementary Methods). Overall, soil chemistry and climatic variables were insignificant predictors of community-scale variation in fungal gene dissimilarity (all Pfam domains: Supplementary Figure 4). We further studied composition-function linkages, finding that fungal communities and functional gene composition were significantly correlated after accounting for geographic distances among samples (Partial Mantel r = 0.31, P = 0.007; Fig. 1c).

**Figure 2:**
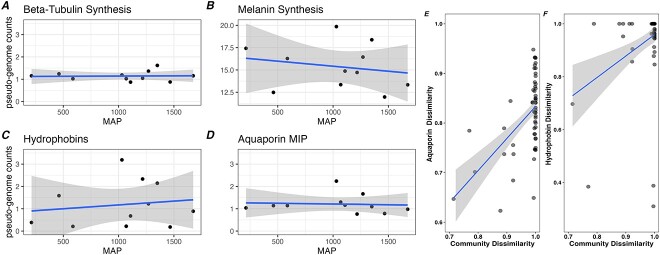
(A-D). Normalized abundance of gene family counts across the precipitation gradient: Mean annual precipitation (MAP). In order to account for variation in sequencing depth and the compositional nature of the metagenomic data, we performed a procedure that normalizes gene counts by those encoding the near single-copy gene Asparaginase. This additive log-ratio procedure, allows for the calculation of pseudo-genome counts for key gene families. Lines represent linear splines with 95% confidence intervals. (E-F). Correlations between EMF community dissimilarity (Bray–Curtis), and aquaporin and Hydrophobin sequence dissimilarity based on Pfam homology.

We hypothesized that trees occurring in comparatively drier soils would host EMF enriched in genes related to drought stress and water acquisition. Although individual gene families involved in melanin synthesis exhibited hypothesized trends across the precipitation gradient, these relationships were statistically insignificant (Supplementary Figure 5). We studied the cumulative abundance of genes involved in melanin synthesis, aquaporins, trehalose synthases, hydrophobins, respectively, as well as other genes encoding abundant carbohydrate active enzymes (CAZymes). Overall, we detected no statistically significant shifts in gene abundances for any gene category (Fig. 2; Supplementary Fig. 6). Because soil pH was a strong predictor of EMF community composition, we additionally studied correlations in soil pH and abundance of targeted gene families; we observed broadly similar but overall insignificant relationships (Supplementary Fig. 7).

While the above analyses measured gene abundances on a statistically derived genome level, we additionally reasoned that *P. trichocarpa* occurring in dry soils would host a greater total abundance of EMF genes involved in drought stress tolerance. Estimating the net abundance of EMF genes involved in water relations captures relative plant investment and reliance on the EMF community. To estimate plant investment in EMF across this gradient we weighted metagenomic gene-counts by the percentage of root-length colonized by EMF (Supplementary Fig. 8); this weighting procedure serves as an estimate of the cumulative biomass of EMF on roots [3]. We detected strong negative relationships in the weighted abundance of genes involved in melanin synthesis (*P* = .03, *R*^2^ = 0.36), aquaporins (*P* = .02, *R*^2^ = 0.43), trehalose synthases (*P* = .03, *R*^2^ = 0.35), alpha amylase (PF00128; *P* = .03, R^2^ = 0.35), melanin synthases (*P* = .02, R^2^ = 0.36), but not fungal hydrophobins (*P* = .86) (Supplementary Fig. 8). Similar patterns were observed when weighted gene counts were regressed against soil pH (Supplementary Fig. 9). We acknowledge that this weighting-procedure is coarse, and that the effect of MAP for individual target gene families must be compared with genes that serve as a statistical null. In this case, Beta-tubulins, a gene at near-single copy in fungal genomes and plausibly under minimal ecological selective pressure across this soil gradient, exhibited similar relationships as observed for functional genes (*P* = .02, *R*^2^ = 0.40). This therefore limits understanding of the significance of the cumulative abundance of these gene families in plant water relations. Sampling from other host-trees would be useful to help understand the generality of the patterns observed here.

Finally, we investigated potential intra-genetic variation in functional genes by focusing on gene sequence homology. Using a partial mantel test, we identified aquaporin (Mantel *r* = 0.50, *P* = .001) and hydrophobin (Mantel *r* = 0.33, *P* = .023) sequence dissimilarity (Bray–Curtis) as positively correlated with EMF community dissimilarity (Fig. 2E and F). Consequentially, the functioning of aquaporins or hydrophobins could co-vary with EMF communities across the precipitation gradient irrespective of shifts in gene abundances. Additional targeted gene families either did not have sufficient data for calculation of distance matrices or exhibited insignificant relationships. Further functional analysis is required to understand if fungal aquaporins or hydrophobins vary in their activity across this gradient.

## Conclusions

EMF communities associated with *P. trichocarpa* exhibited large compositional shifts across a natural precipitation gradient. Moreover, *P. trichocarpa* root-systems were significantly more colonized in drier soils. Despite distinct EMF communities occurring in drier soils, the abundance of genes hypothesized to be involved in drought stress tolerance was relatively invariant. One scenario is that EMF differentially express genes putatively involved in drought-stress tolerance despite possessing a similar core repertoire. In addition, our results demonstrating significant variation in sequence homology for aquaporin and hydrophobin genes could be suggestive of differences in the functioning of these genes across the studied precipitation gradient. In both cases metagenomic profiling would be unable to differentiate amongst these possibilities. Our results therefore suggest that meta-transcriptomic, or metabolomic profiling could be necessary to infer the physiological attributes of ECM drought stress tolerance and plant water uptake [11, 20]. Finally, due to the small number of communities studied here, additional sampling is necessary to infer the role of MAP and soil water availability in structuring these communities. Moreover, we acknowledge that the patterns observed here are challenging to disentangle from soil variables like pH.

## Supplementary Material

Supplementary_ISME_Comms_2024_ycae031

## Data Availability

ITS amplicon sequence data are archived in SRA SUB13575173, and bioproject accession PRJNA987748. Metagenomic data is available through JGI, IMG # 10.46936/10.25585/60000790. No restrictions to data utilization.
