# Supplementary material for: Potential for functional divergence in ectomycorrhizal fungal communities across a precipitation gradient
Source: ISME Commun. 2024 Mar 4;4(1):ycae031. doi: 10.1093/ismeco/ycae031 (PMC10960952; doi:10.1093/ismeco/ycae031)
Supplement: Supplementary_ISME_Comms_2024_ycae031 [file supplementary_isme_comms_2024_ycae031.docx]

Supplementary Information for: *Potential for functional divergence in Ectomycorrhizal fungal communities across a precipitation gradient*

***Supplementary Methods:***

***Site description and field sampling***

Sites were identified as natural stands dominated by *Populus* *trichocarpa* (>80% forest basal area) to reduce the influence of other plant species. In July and August of 2017, samples were collected from the base of five individual mature trees to a depth of 10cm. Previous studies conducted in these sites have focused on foliar endophyte communities of *Populus trichocarpa [1].* Each of the five individuals were sampled at least 10m apart to avoid subsampling genetic clones. Samples from each tree were then composited in the field. To avoid sampling roots from other plant species or *Populus* individuals, we excavated small areas to perform root tracing back to the focal tree trunk while collecting roots. Soils from immediately adjacent to the root-core were collected for soil chemical analysis. Root-cores were stored on ice and transported to the lab. Definitive ectomycorrhizal fungal root-tips were removed under a dissecting scope after visual confirmation of ectomycorrhizal mantle and high turgor. Ectomycorrhizal root-tip samples were sequentially washed of adhering soil particles to remove potential saprotrophic and bacterial contaminants.

***DNA Extraction and Bioinformatic Workflow*:**

DNA was extracted from at least ten, composited colonized root-tips using a Qiagen DNeasy Plant Mini Kit following manufacturers protocol. Extraction replicates were combined for each sample and DNA recovery was assessed using gel electrophoresis and a Qubit spectrophotometer. Samples were split into DNA pools for amplicon sequencing and shotgun-metagenomic sequencing. Amplicons were generated using PCR of the ITS1 using the ITS1f-ITS2 primer pair [2]. Sequences were generated using Illumina Miseq (2 x 250). Due to the generally low quality of reverse reads, only forward reads were retained for Illumina sequences. Single read approaches have been found to be highly accurate in recovering mock fungal communities [3]. Illumina-sequenced amplicon reads were processed using the DADA2 pipeline [4] with the following quality filtering parameters: *maxN* = 0, *maxEE* = 8, *truncQ* = 2, *minLen* = 50. ASVs were assigned taxonomy using the UNITE dynamic database (v9) [5]. Due to variation in sequencing depth across samples, samples were rarefied to 768 sequences.

Metagenomic sequence libraries were constructed and sequenced at JGI using Illumina NovaSeq. We employed the JGI IMG pipeline to filter and annotate fungal reads, whilst removing plant and bacterial sequences [6]. 11 samples passed quality control (Q=20).

Across all samples approximately 23.5% of assembled contigs (SD = 5.16) were assigned to fungi. Sequences were assembled using SPAdes [7], and annotated using Prodigal [8] using standard parameters. All contigs with best hits from prokaryotes or plants were discarded. Remaining sequences were again searched against Mycocosm and Phytozome (JGI's fungal and plant databases) using MMseqs2 [9] and those matching to plants were discarded. On average a total of 366,338,399 (SD= 3.36) reads were assigned to fungi in each sample. Contigs were assembled and those assigned to Agaricomycotina and Pezizomycotina were retained in order to primarily focus on EM forming fungi. Filtered contigs were annotated against the Pfam database using HMMER, with standard parameters [10, 11].The compositional nature of the data was accounted for by normalizing sequence counts against Asparaginase gene counts which are thought to be at near sing-copy abundance [12] and by log-transforming these ratios. This approach is identical to an additive log-ratio transformation.

***Soil properties***

All soil related properties were measured at A and L labs (<https://al-labs-west.com>), in Modesto CA, USA. Briefly, soil organic matter was calculated by percentage loss on ignition (LOI). Soil pH was measured using a 1:1 soil:water suspension. Further details on analytical procedures can be obtained on the website. Bioclim data was collected using information derived from GPS coordinates [13].

**Data Analysis:**

Permutational analysis of variance (PERMANOVA) in the package *vegan* *v.*2.6-4 was used to study soil and climatic drivers of ectomycorrhizal fungal dissimilarity using Bray-Curtis distances. Community and functional variation was visualized using ordination methods (NMDS). A partial mantel test was employed to study the relationship between Taxonomic and Functional dissimilarity while including geographic distances among sites. We calculated geographic distances between site pairs using *geodist v.*0.08. After identifying key gene families potentially involved in drought stress, we searched for relevant genes in annotated metagenomes using Pfam ID’s. We performed linear regression of target gene counts against either mean annual precipitation or pH. In order to study most abundant CAZy we first calculated the most abundant CAZy genes across all samples, selecting those with at least 20 total hits. We plotted the abundance of target gene counts across either a gradient of mean annual precipitation or soil pH. For each target Pfam domain, we calculated a metric of sequence dissimilarity based on sequence homology, using Bray-Curtis distances. We used partial mantel tests to study the correlation between sequence dissimilarity and taxonomic dissimilarity.

Supplementary References.

1. Barge EG, Leopold DR, Peay KG, Newcombe G, Busby PE. Differentiating spatial from environmental effects on foliar fungal communities of Populus trichocarpa. *Journal of Biogeography* 2019; **46**: 2001–2011.

2. Smith DP, Peay KG. Sequence Depth, Not PCR Replication, Improves Ecological Inference from Next Generation DNA Sequencing. *PLOS ONE* 2014; **9**: e90234.

3. Pauvert C, Buée M, Laval V, Edel-Hermann V, Fauchery L, Gautier A, et al. Bioinformatics matters: The accuracy of plant and soil fungal community data is highly dependent on the metabarcoding pipeline. *Fungal Ecology* 2019; **41**: 23–33.

4. Callahan BJ, McMurdie PJ, Rosen MJ, Han AW, Johnson AJA, Holmes SP. DADA2: High-resolution sample inference from Illumina amplicon data. *Nature Methods* 2016; **13**: 581–583.

5. Nilsson RH, Larsson K-H, Taylor AFS, Bengtsson-Palme J, Jeppesen TS, Schigel D, et al. The UNITE database for molecular identification of fungi: handling dark taxa and parallel taxonomic classifications. *Nucleic Acids Res* 2019; **47**: D259–D264.

6. Chen I-MA, Chu K, Palaniappan K, Pillay M, Ratner A, Huang J, et al. IMG/M v.5.0: an integrated data management and comparative analysis system for microbial genomes and microbiomes. *Nucleic Acids Res* 2019; **47**: D666–D677.

7. Bankevich A, Nurk S, Antipov D, Gurevich AA, Dvorkin M, Kulikov AS, et al. SPAdes: A New Genome Assembly Algorithm and Its Applications to Single-Cell Sequencing. *Journal of Computational Biology* 2012; **19**: 455–477.

8. Hyatt D, Chen G-L, LoCascio PF, Land ML, Larimer FW, Hauser LJ. Prodigal: prokaryotic gene recognition and translation initiation site identification. *BMC Bioinformatics* 2010; **11**: 119.

9. Steinegger M, Söding J. MMseqs2 enables sensitive protein sequence searching for the analysis of massive data sets. *Nature Biotechnology* 2017; **35**: 1026–1028.

10. Mistry J, Chuguransky S, Williams L, Qureshi M, Salazar GA, Sonnhammer ELL, et al. Pfam: The protein families database in 2021. *Nucleic Acids Research* 2021; **49**: D412–D419.

11. Potter SC, Luciani A, Eddy SR, Park Y, Lopez R, Finn RD. HMMER web server: 2018 update. *Nucleic Acids Research* 2018; **46**: W200–W204.

12. Quinn TP, Erb I, Richardson MF, Crowley TM. Understanding sequencing data as compositions: an outlook and review. *Bioinformatics* 2018; **34**: 2870–2878.

13. Fick SE, Hijmans RJ. WorldClim 2: new 1-km spatial resolution climate surfaces for global land areas. *International Journal of Climatology* 2017; **37**: 4302–4315.


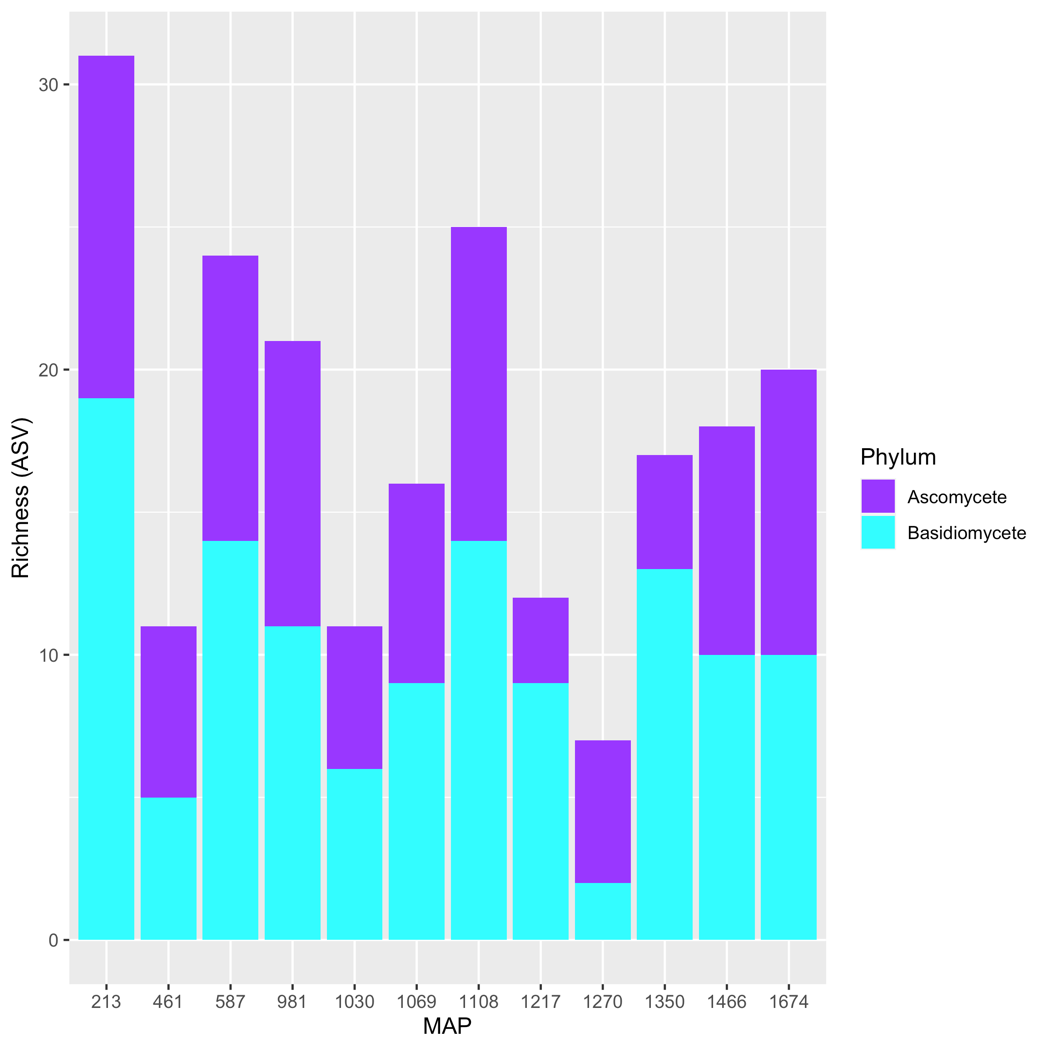


**Supplementary Figure 1.** The richness of Ascomycete and Basidiomycete fungi in each root-tip sample as measured using fungal amplicon sequence variants (ASV), across the precipitation gradient. X-axis displays mean annual precipitation (mm; MAP).


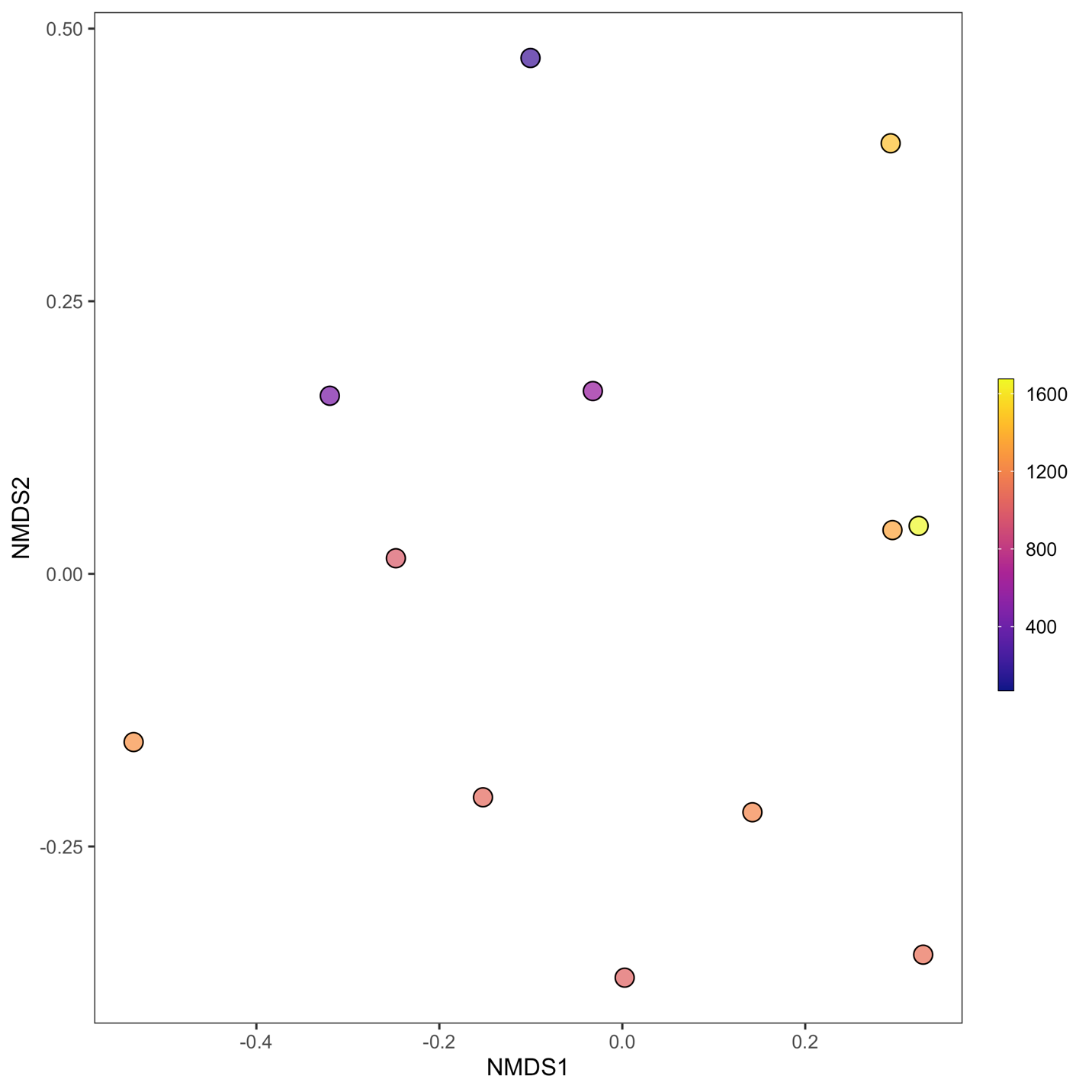


**Supplementary Figure 2**. NMDS ordination of EMF root-tip communities (Bray-Curtis distances), colored by mean annual precipitation (MAP; mm).


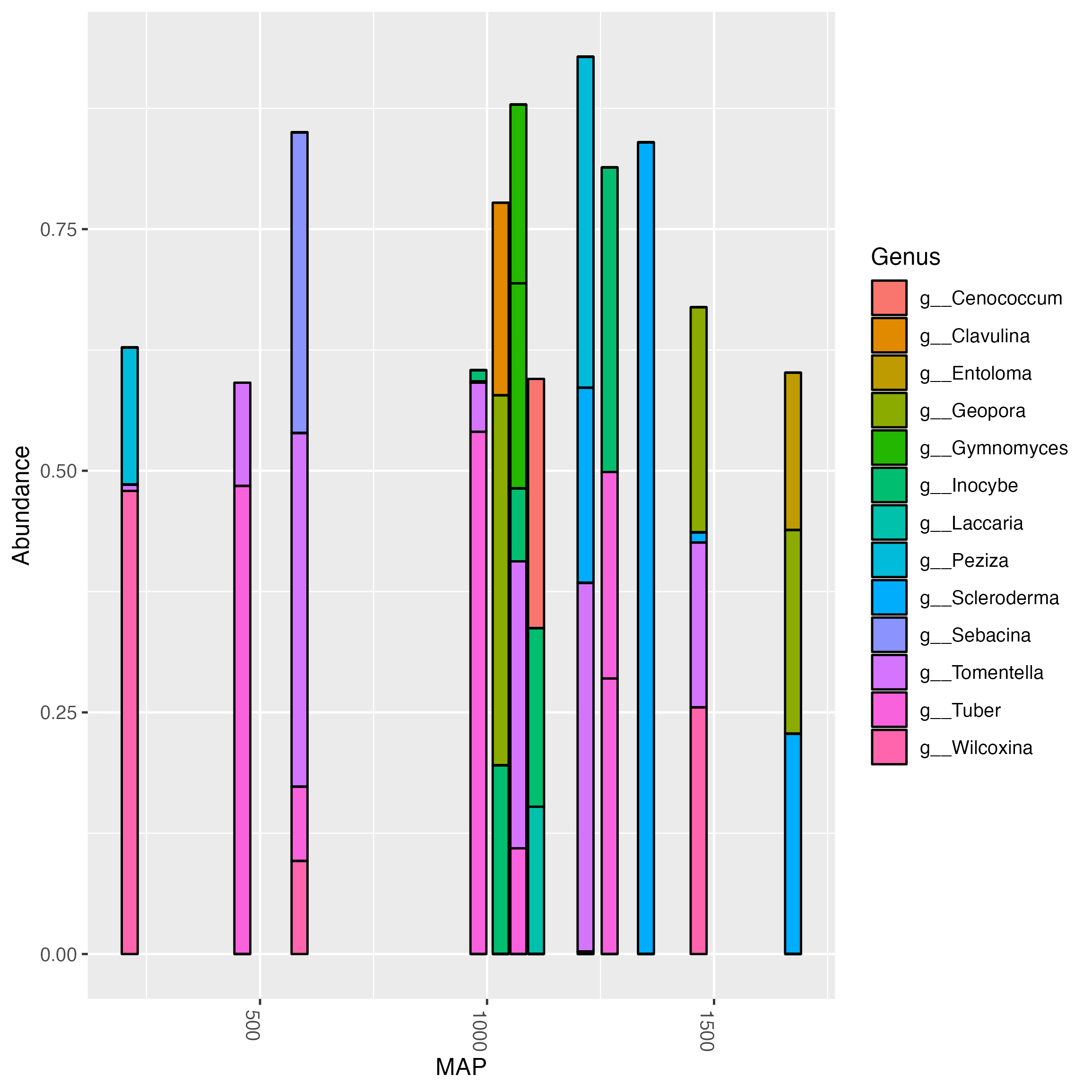


**Supplementary Figure 3.** The relative abundance of the top 25 most abundant EMF taxa present in each sample, arranged by increasing mean annual precipitation (MAP; mm).

**
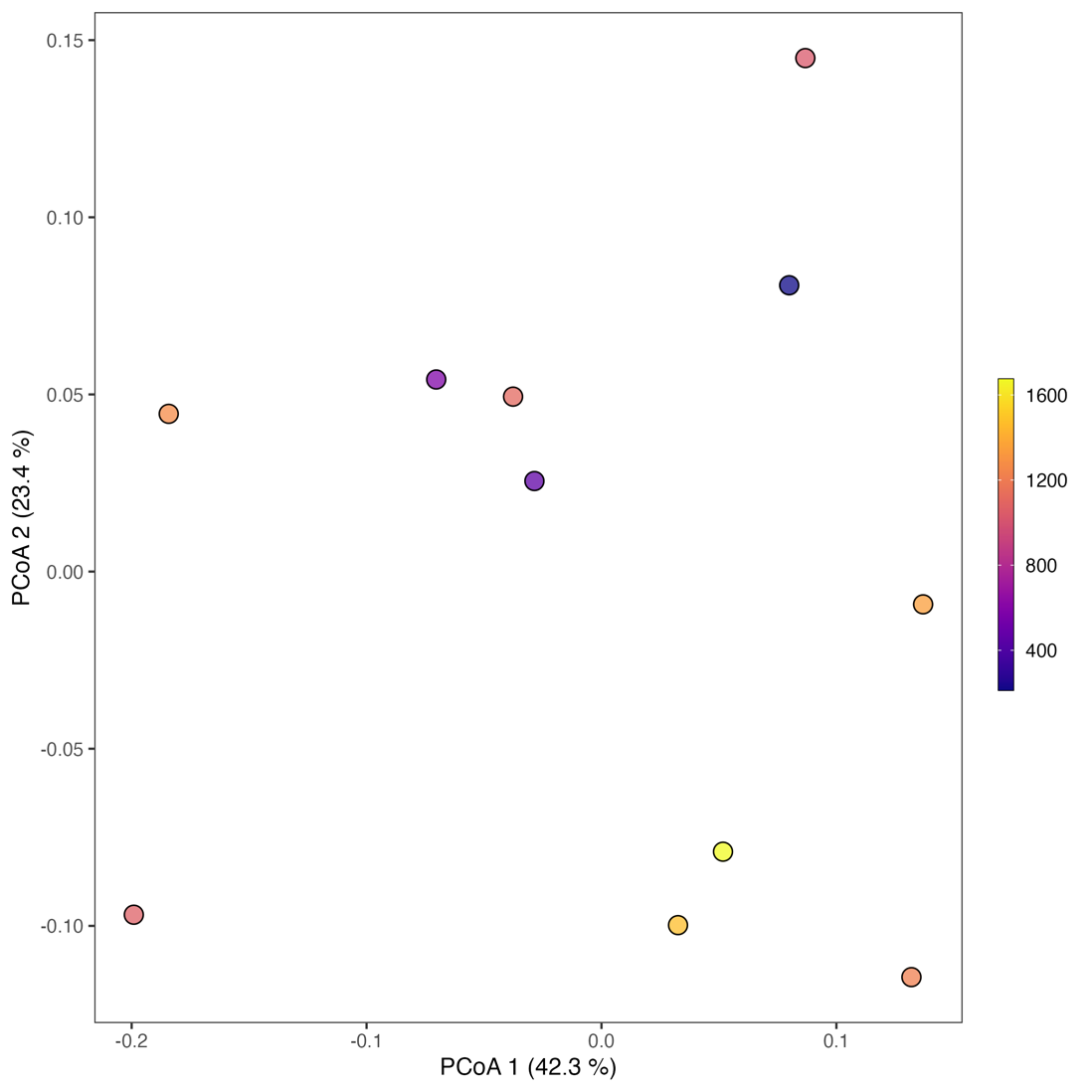
**

**Supplementary Figure 4).** PCoA of EMF communities based on Pfam annotations including all gene families (Bray-Curtis distances). Points are coloured by mean annual precipitation (MAP; mm).


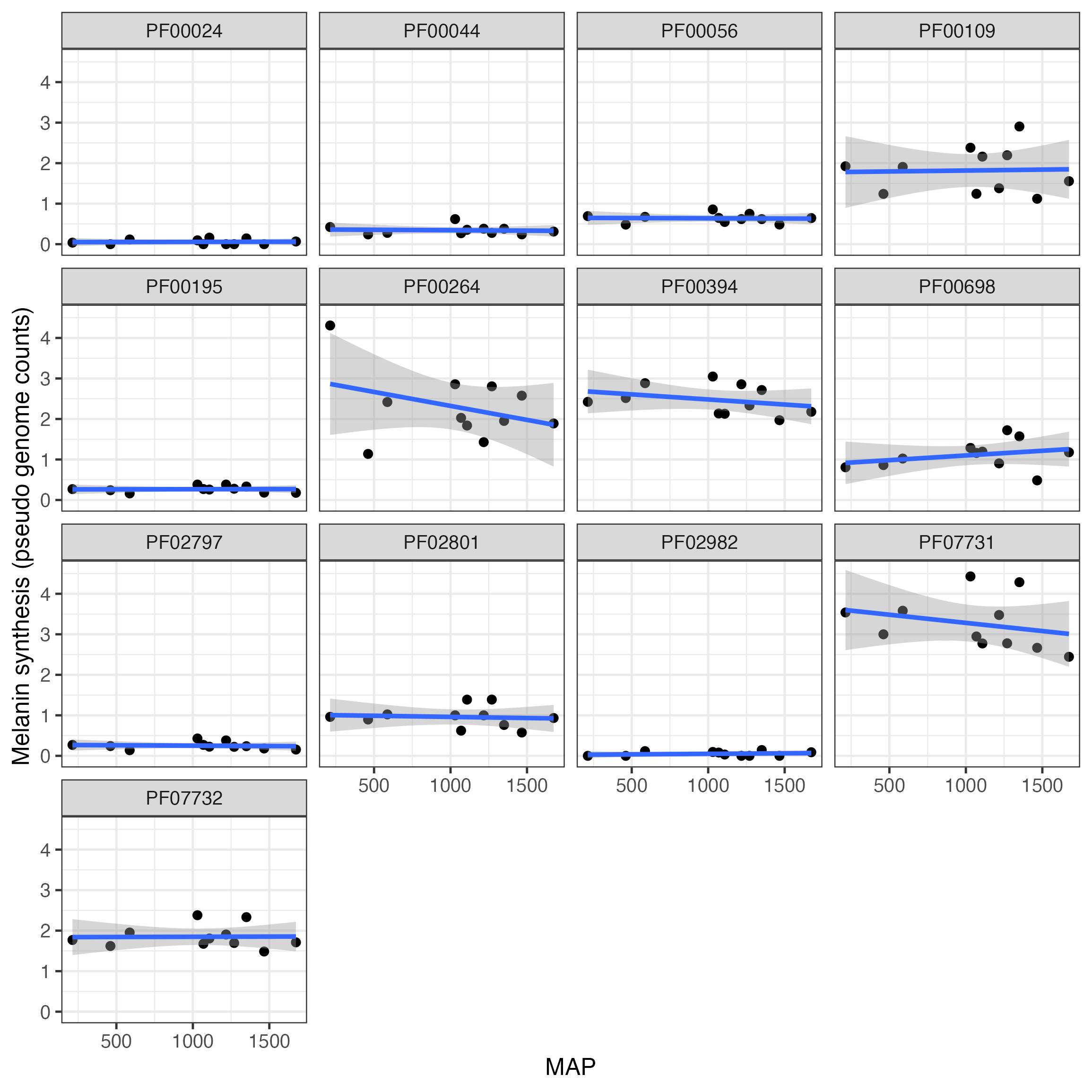


**Supplementary Figure 5).** The abundance of Pfam gene families involved in melanin synthesis regressed against mean annual precipitation (MAP;mm). All relationships are statistically insignificant, but trend lines are shown, representing linear splines with 95% confidence intervals. These Pfam represent the breadth of genes involved in Melanin synthesis. Gene ID’s are described in Supplementary Table 3.

**
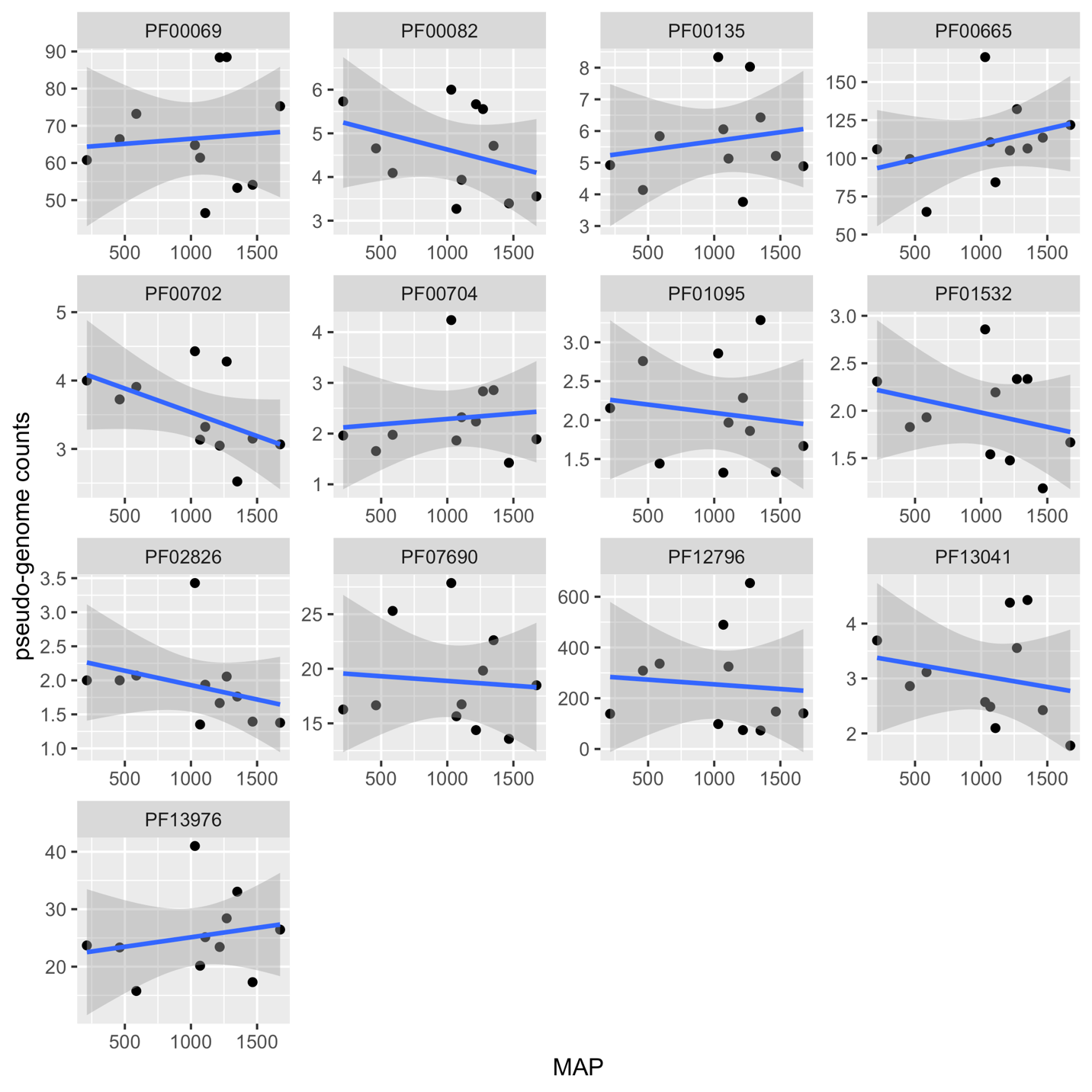
**

**Supplementary Figure 6.** Carbohydrate active enzymes (CAZy), that are present in at least a total of 20 hits across samples, regressed against mean annual precipitation (MAP). All relationships are statistically insignificant. See Supplementary Table 3 for description of individual Pfam ID’s. Lines represent linear splines with 95% confidence intervals.


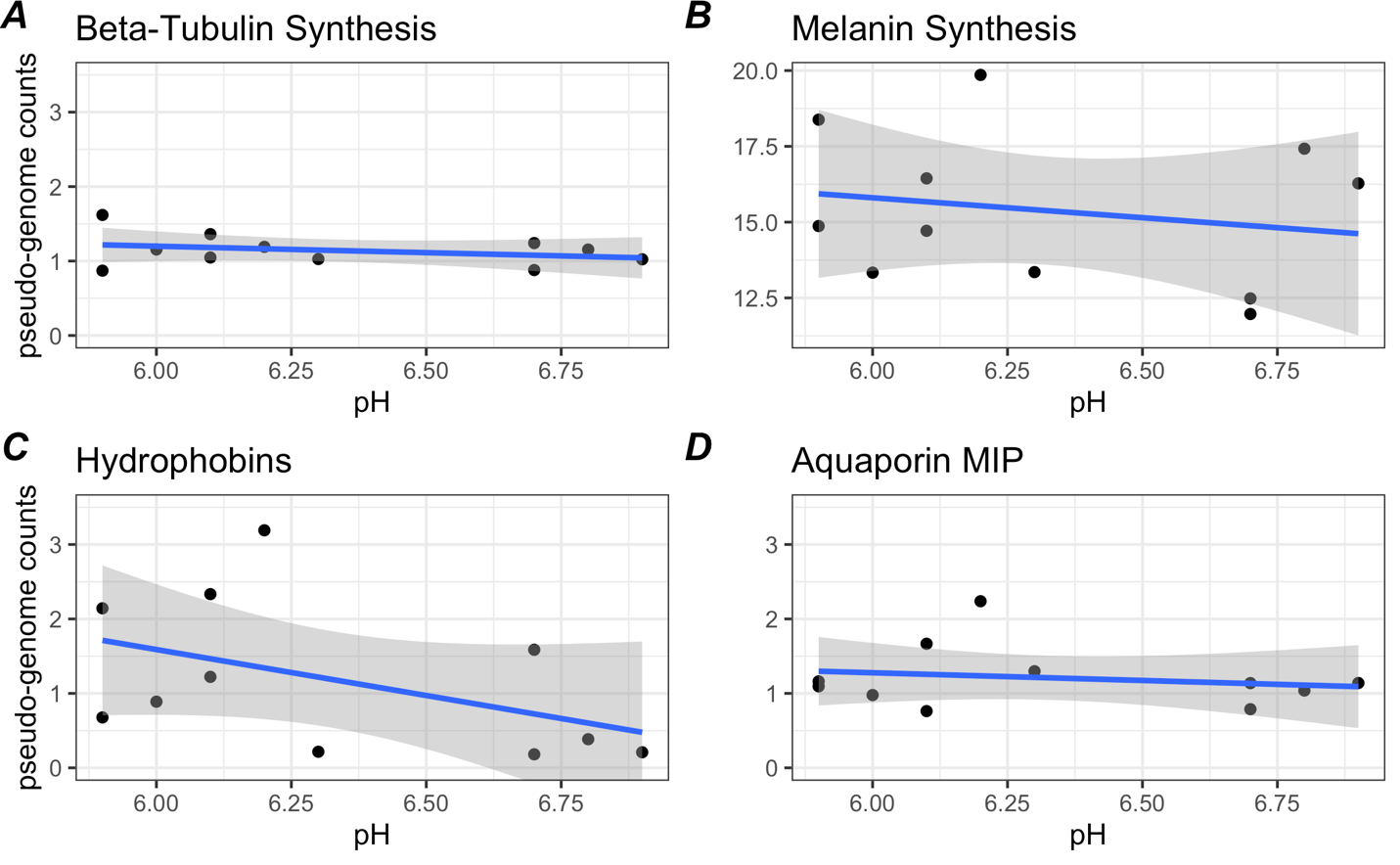


**Supplementary Figure 7).** The abundance of gene counts regressed against the soil pH gradient. Beta-Tubulin Synthesis (*P =*0.36), Melanin Synthesis (*P =* 0.56), Hydrophobins (*P=* 0.16), Aquaporins (*P=*0.58). Lines represent linear splines with shaded 95% confidence intervals.


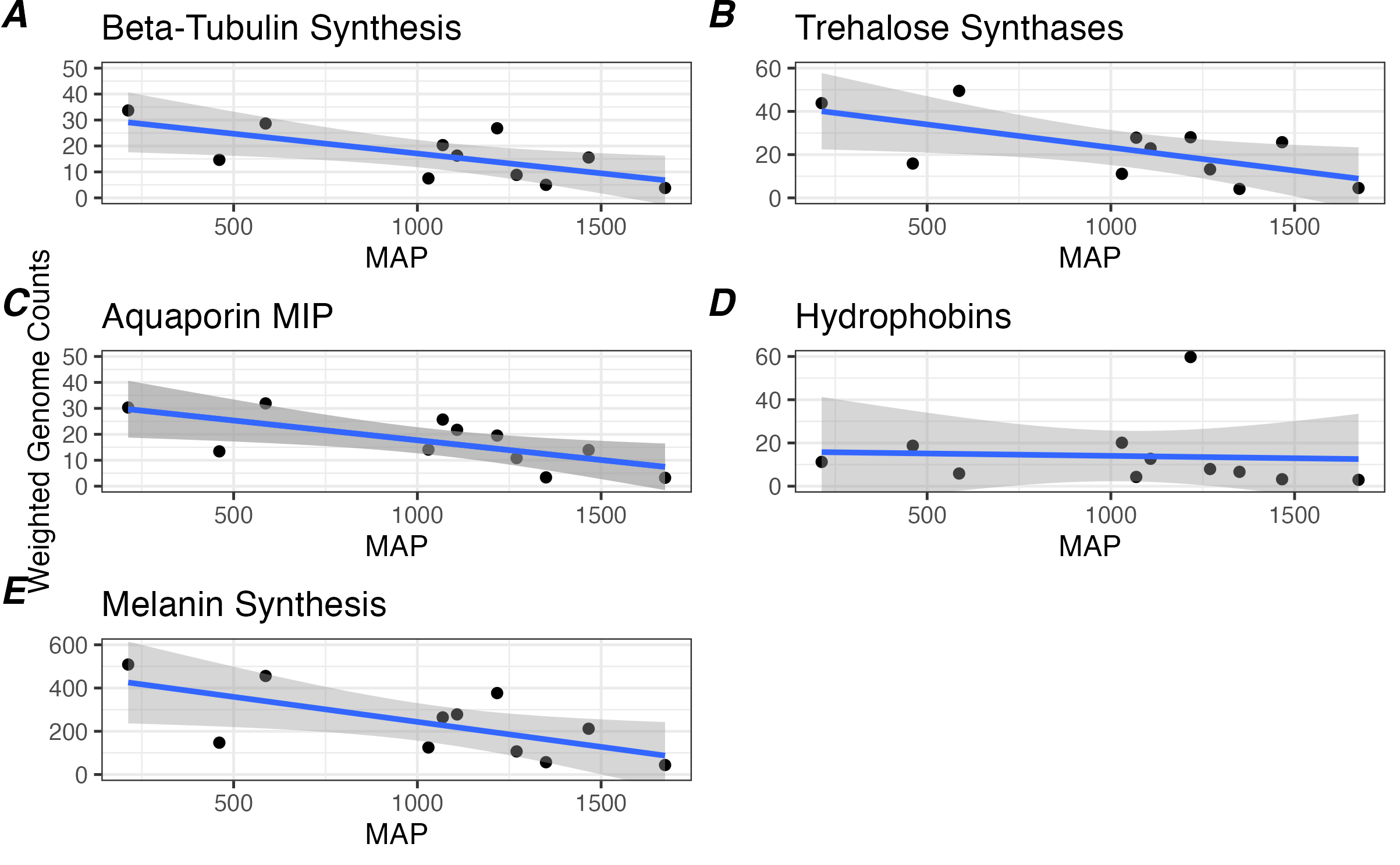


**Supplementary Figure 8).** Linear regression of select gene families across the precipitation gradient. Gene counts are weighted by the relative EMF colonization of each root-system. A). Beta-Tubulin Synthesis (*P= 0.02,* R^2^= 0.40*).* B). Trehalose Synthases (*P =* 0.03, R^2^ = 0.35 *).* C). Aquaporins (*P* = 0.02, R^2^=0.43). Hydrophobins (*P* = 0.61), Melanin Synthesis (*P*= 0.02, R^2^ =0.36). Lines represent linear splines with shaded 95% confidence intervals.


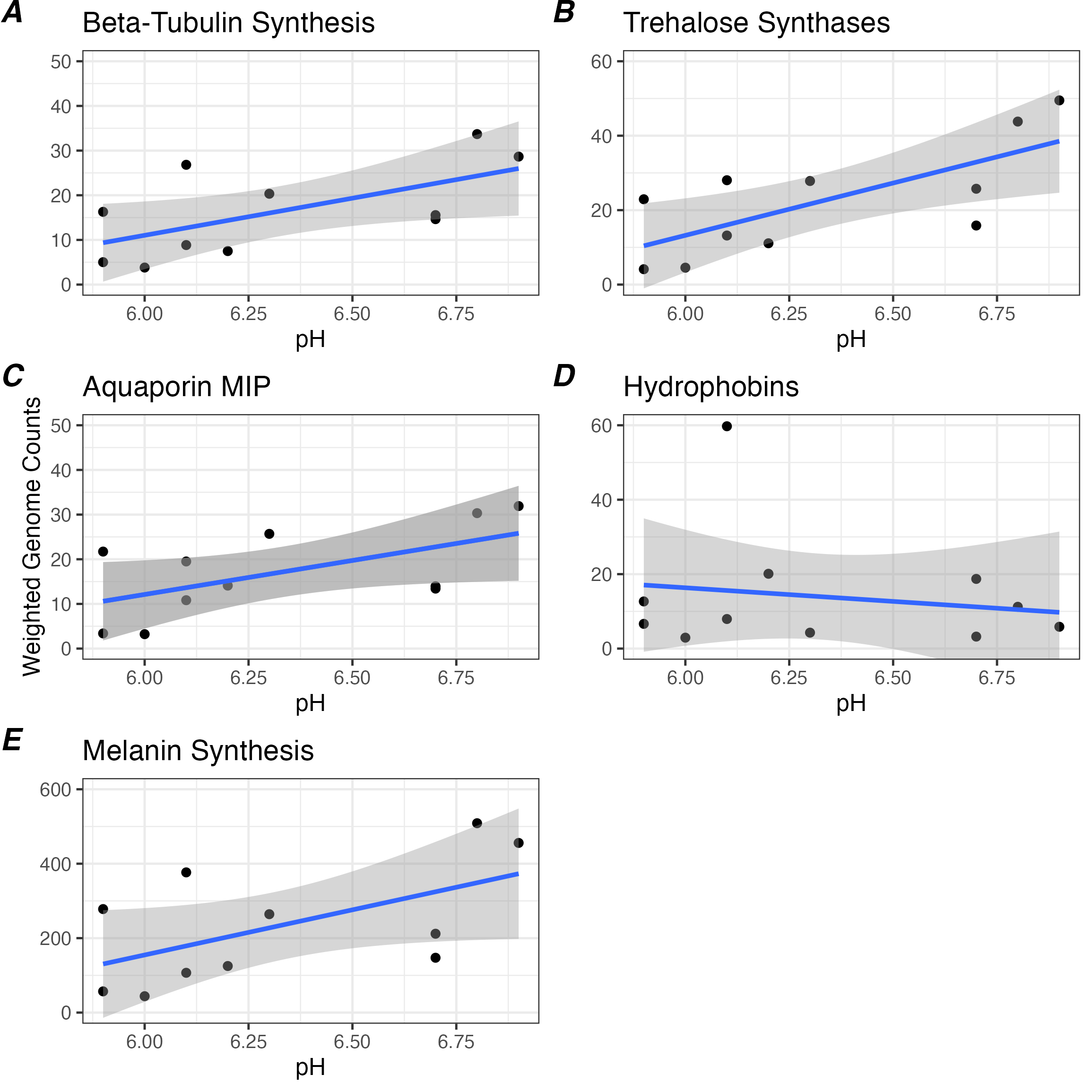


**Supplementary Figure 9).** Linear regression of select gene families across the pH gradient. Gene counts are weighted by the relative EMF colonization of each root-system. A. Beta-Tubulin Synthesis (*P= 0.* 040*,* R^2^= 0.32*).* B). Trehalose Synthases (*P =* 0.01, R^2^ = 0.46 *).* C). Aquaporins (*P* = 0.02, R^2^=0.43). Hydrophobins (*P* = 0.61), Melanin Synthesis (*P*= 0. 06, R^2^ =0.28). Gene counts are weighted by the relative colonization of each root-system. Lines represent linear splines with shaded 95% confidence intervals.

**Table 1.** Predictors of ectomycorrhizal fungal community dissimilarity (Bray-curtis distances). Results from PERMANOVA.

|  | Df | SumOfSqs | R^2^ | F | P |
| --- | --- | --- | --- | --- | --- |
| %Carbon | 1 | 0.54 | 0.10 | 1.26 | 0.10 |
| N_release | 1 | 0.40 | 0.08 | 0.95 | 0.60 |
| pH | 1 | 0.67 | 0.13 | 1.57 | 0.01 |
| MAT | 1 | 0.42 | 0.08 | 1.00 | 0.49 |
| MAP | 1 | 0.54 | 0.11 | 1.27 | 0.06 |
| Residual | 6 | 2.55 | 0.50 |  |  |
| Total | 11 | 5.13 | 1.00 |  |  |

**Table 2**. Predictors of Pfam dissimilarity in Ectomycorrhizal fungal communities (Bray-curtis distances). Results from PERMANOVA

|  | Df | SumOfSqs | R^2^ | F | P |
| --- | --- | --- | --- | --- | --- |
| MAP | 1 | 0.035 | 0.11 | 1.16 | 0.32 |
| pH | 1 | 0.024 | 0.08 | 0.81 | 0.58 |
| %C | 1 | 0.054 | 0.17 | 1.78 | 0.11 |
| N_release | 1 | 0.14 | 0.045 | 0.44 | 0.89 |
| MAT | 1 | 0.017 | 0.06 | 0.57 | 0.76 |
| Residual | 6 | 0.181 | 0.58 |  |  |
| Total | 10 | 0.311 | 1 |  |  |

**Table 3**. Pfam domains analyzed in the present study, and their encoded function.

| **Pfam domain** | **Encoded function** |
| --- | --- |
| PF00230 | Aquaporin |
| PF01185 | Hydrophobin |
| PF06766 | Hydrophobin |
| PF02797 | Melanin Synthase (Chalcone synthesis) |
| PF00195 | Melanin Synthase (Chalcone synthesis) |
| PF00698 | Melanin Synthase (Acyl transferase) |
| PF02982 | Melanin Synthase ( [Scytalone dehydratase](https://www.ebi.ac.uk/interpro/protein/reviewed/P56221)) |
| PF00044 | Melanin Synthase ([Glyceraldehyde 3-phosphate dehydrogenase](https://www.ebi.ac.uk/interpro/entry/pfam/PF00044/logo/)) |
| PF00056 | Melanin Synthase ([lactate/malate dehydrogenase)](https://www.ebi.ac.uk/interpro/entry/pfam/PF00056) |
| PF00394 | Melanin Synthase (Multicopper oxidase) |
| PF00024 | Melanin Synthase (PAN domain) |
| PF07731 | Melanin Synthase (Multicopper oxidase) |
| PF07732 | Melanin Synthase (Multicopper oxidase) |
| PF00264 | Melanin Synthase (Tyrosinase) |
| PF03723 | Melanin Synthase ([Hemocyanin)](https://www.ebi.ac.uk/interpro/entry/pfam/PF03723) |
| PF00372 | Melanin Synthase (Hemocyanin) |
| PF03722 | Melanin Synthase (Hemocyanin) |
| PF02801 | Melanin Synthase ( Beta-ketoacyl synthase) |
| PF00109 | Melanin Synthase (Beta-ketoacyl synthase) |
| PF00128 | Trehalose Synthase |
| PF16657 | Trehalose Synthase |
| PF00128 | Alpha Amylase |
| PF12796 | Ankyrin_repeats_(3_copies) |
| PF00665 | Integrase_core_domain |
| PF00069 | Protein_kinase_domain |
| PF13976 | GAG-pre-integrase_domain |
| PF07690 | Major_Facilitator_Superfamily |
| PF00135 | Carboxylesterase_family |
| PF00082 | Subtilase_family |
| PF00702 | haloacid_dehalogenase-like_hydrolase |
| PF13041 | PPR_repeat_family |
| PF00704 | Glycosyl_hydrolases_family_18_GH18 |
| PF01095 | Pectinesterase |
| PF01532 | Glycosyl_hydrolase_family_47_GH47 |
| PF02826 | D-isomer_specific_2-hydroxyacid_dehydrogenase,_NAD_binding_domain |

**Table 4**: Study Sites, with relevant metadata. DBH (Diamater at breast height). ECM colonization is measured using grid-intersect method. Paramaters are reported in Supplementary Methods.
